# A systematic review of volumetric image guidance in proton therapy

**DOI:** 10.1007/s13246-023-01294-9

**Published:** 2023-06-29

**Authors:** Mitchell Herrick, Scott Penfold, Alexandre Santos, Kevin Hickson

**Affiliations:** 1 Department of Radiation Oncology, Royal Adelaide Hospital, Adelaide, Australia; 2 Department of Physics, University of Adelaide, Adelaide, Australia; 3 Australian Bragg Centre for Proton Therapy and Research, Adelaide, Australia; 4 SA Medical Imaging, Adelaide, Australia; 5 University of South Australia, Allied Health & Human Performance, Adelaide, Australia

**Keywords:** Image-guided proton therapy, Volumetric image guidance, In-room imaging, Adaptive proton therapy

## Abstract

In recent years, proton therapy centres have begun to shift from conventional 2D-kV imaging to volumetric imaging systems for image guided proton therapy (IGPT). This is likely due to the increased commercial interest and availability of volumetric imaging systems, as well as the shift from passively scattered proton therapy to intensity modulated proton therapy. Currently, there is no standard modality for volumetric IGPT, leading to variation between different proton therapy centres. This article reviews the reported clinical use of volumetric IGPT, as available in published literature, and summarises their utilisation and workflow where possible. In addition, novel volumetric imaging systems are also briefly summarised highlighting their potential benefits for IGPT and the challenges that need to be overcome before they can be used clinically.

## Introduction

Image guidance in radiation therapy aims to quantify and mitigate the effect of inter- and intrafractional patient geometry variation. It is a critical aspect in modern photon and proton therapy treatment processes and has allowed for a reduction in treatment margins. Until relatively recently, proton therapy centres have typically relied on 2D x-ray imaging for image guidance, using bony anatomy or implanted fiducial markers to verify patient alignment prior to treatment. 2D imaging, however, cannot be used to assess soft tissue geometry, and cannot be used for dose tracking or supporting adaptive proton therapy workflows. For these applications, a form of in-room volumetric imaging is required. It is generally acknowledged that the implementation of image guided proton therapy (IGPT) has lagged behind image guided radiation therapy (IGRT) with x-rays [1]. Furthermore, because of the greater diversity in proton therapy vendors, the type of volumetric imaging utilised in proton therapy varies significantly between centres.

This review reports on the use of volumetric imaging systems for clinical IGPT, as described in published literature. Based on the information available, we summarise the utilisation and associated workflows of the various IGPT systems. Finally, we will briefly discuss novel systems that may be translated to clinical use in the future.

## Methodology

### Search parameters

A systematic review was undertaken to assess the status of clinically used in-room volumetric imaging systems in proton therapy centres. Beginning in November 2021, several searches of the PubMed database were conducted to generate a list of articles to review. Search terms included *(in-room imaging) AND (proton therapy), (adaptive proton therapy)*, as well as *(proton therapy OR particle therapy) AND (image guidance OR image guided OR IGPT)* to refine the search. From each search, a combined total of 734 articles were gathered.

### Screening

**Fig. 1:**
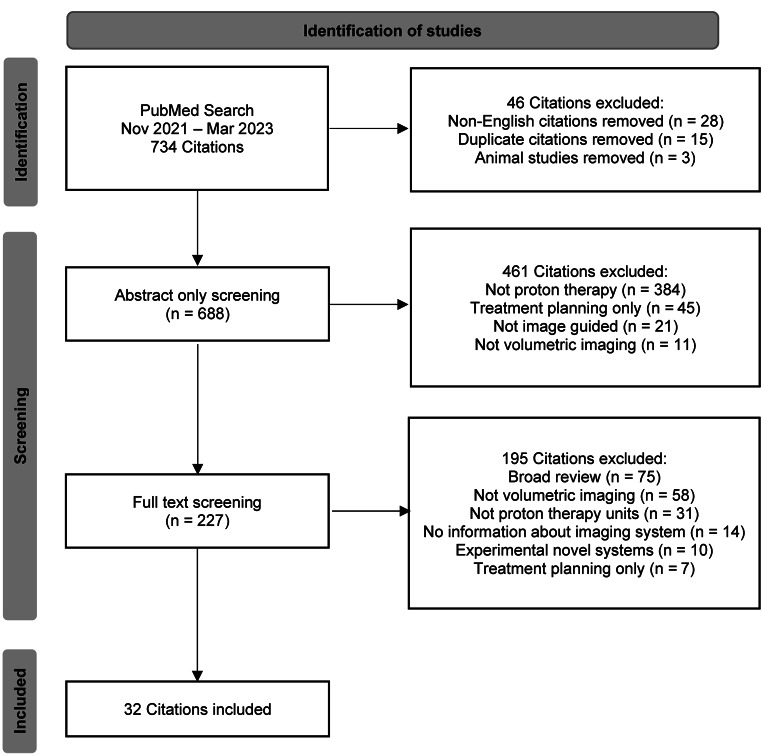
Flowchart of the screening process

Articles were initially reviewed by abstract and retained based on relevance to volumetric image guidance. Articles that had some ambiguities regarding image guidance were accepted for the full-text review. Articles about 2D kV image guidance or CT-simulation for initial treatment planning imaging were excluded. In addition, articles that had no relevance to proton therapy or were not in English were also excluded. During the full-text review, articles were only included if they had some form of evidence regarding clinical usage of volumetric image guidance for proton therapy. Articles that contained information regarding non-clinical experimental systems were also excluded. In addition, articles that described a future implementation of volumetric imaging (such as technical notes) were excluded. A more detailed screening summary can be seen in Fig. 1.

## Results

After screening, thirty-two articles were found to reference some form of 3D volumetric image guidance in a proton therapy centre. Each study was grouped in one of the following categories:

1. CT group: Reported image guidance protocols that use some form of fan beam CT imaging modality. The method of usage and modality type of each study has been tabulated in Table 1.
  **Table 1:** Studies that report the use of a CT system for IGPT.

Author	CT imaging modality type	Treatment isocentre imaging	Imaging usage	
			Patient positioning	Dose verification
Kamada et al. (1999)	In-room Horizontal CT on rails	Yes*	Yes	Yes
Bolsi et al. (2008)	Fixed out-of-room CT	No	Yes	No
Stuetzer et al. (2016)	In-room CT, no further info	No	Yes	No
Abe et al. (2017)	CT system, no further info**	No	Yes	Yes
Maeda et al. (2018)	In-room CT on rails	No	Yes	No
Maeda et al. (2018)	In-room CT on rails	No	Yes	No
Oliver et al. (2018)	Mobile CT	No	Yes	Yes
Sun et al. (2018)	Mobile CT	No	Yes	Yes
Kurz et al. (2019)	Fixed in-room CT	No	Yes	Yes
Kang et al. (2020)	Mobile CT	No	Yes	No
Maeda et al. (2020)	In-room CT on rails	No	Yes	No
Nenoff et al. (2021)	In-room CT on rails	No	Yes	Yes
Berthold et al. (2021)	Fixed in-room DECT	No	No	Yes
Hongying et al. (2022)	In-room CT on rails	No	Yes	No
Zeidan et al. (2022)	Mobile CT	No	Yes	Yes

* Only when the patient is in a seated treatment position

** Images were taken within 5 min of treatment administration. No details provided if the system in in-room or near room
2. CBCT group: Reported image guidance protocols that use some form of cone beam CT (CBCT) imaging modality. The method of usage and modality type of each study has been tabulated in Table 2.
  **Table 2:** Studies that report the use of a CBCT system for IGPT

Author	CT imaging modality type	Treatment isocentre imaging	Imaging usage	
			Patient positioning	Dose verification
Fattori et al. (2015)	Robotic C-arm CBCT	Yes	Yes	No
Veiga et al. (2016)	Gantry mounted CBCT	Yes	Yes	No
Hua et al. (2017)	Ceiling mounted C-arm robotic CBCT	Yes	Yes	No
Pidikiti et al. (2017)	Onboard CBCT	Yes	Yes	No
Veiga et al. (2017)	Gantry mounted CBCT	Yes	Yes	No
Stock et al. (2018)	Couch mounted CBCT	Yes	Yes	No
Cheon et al. (2020)	Gantry mounted CBCT	Yes	Yes	No
Chilukuri et al. (2020)	Onboard CBCT	Yes	Yes	No
Thummerer et al. (2020)	Onboard CBCT	Yes	Yes	No
Thummerer et al. (2020)	Onboard CBCT	Yes	Yes	No
Davies et al. (2021)	Onboard CBCT	Yes	Yes	No
Uh et al. (2021)	Ceiling mounted C-arm robotic CBCT	Yes	Yes	No
Bondesson et al. (2022)	Onboard CBCT	Yes	Yes	No
Gaikwad et al. (2022)	Onboard CBCT	Yes	Yes	No
Sheikh et al. (2022)	Onboard CBCT	Yes	Yes	No
Stanforth et al. (2022)	Onboard CBCT	Yes	Yes	No
Sharma et al. (2023)	Onboard CBCT	Yes	Yes	No

### CT Systems

Helical CT imaging is the current standard in proton treatment planning for quantitative patient stopping power ratio (SPR) measurements. Thus, using the same imaging modality for IGPT as that used for the initial treatment planning is attractive for many reasons. Using the same modality makes it easier to compare the planning CT (pCT) and pre-treatment CT (ptCT). In addition, image registration between the pCT and ptCT is simplified, and images of similar quality can be obtained allowing the possibility for performing dose calculations on the ptCT. Also, CT systems often have features like 4DCT and dual-energy scans that can be used for ptCT imaging.

However, despite these advantages, one of the greatest drawbacks of in-room (or near-room) CT systems is its inability to image at treatment isocentre. Due to its bulky size, patients are moved away from treatment isocentre and into the CT system’s imaging isocentre for imaging. This introduces additional uncertainty that should be considered due to potential patient motion when moving back to treatment isocentre. In addition to its inability to image at treatment isocentre, CT systems can require a substantial amount of floor space in the treatment room which may not be physically available for some proton therapy centres.

**Fig. 2:**
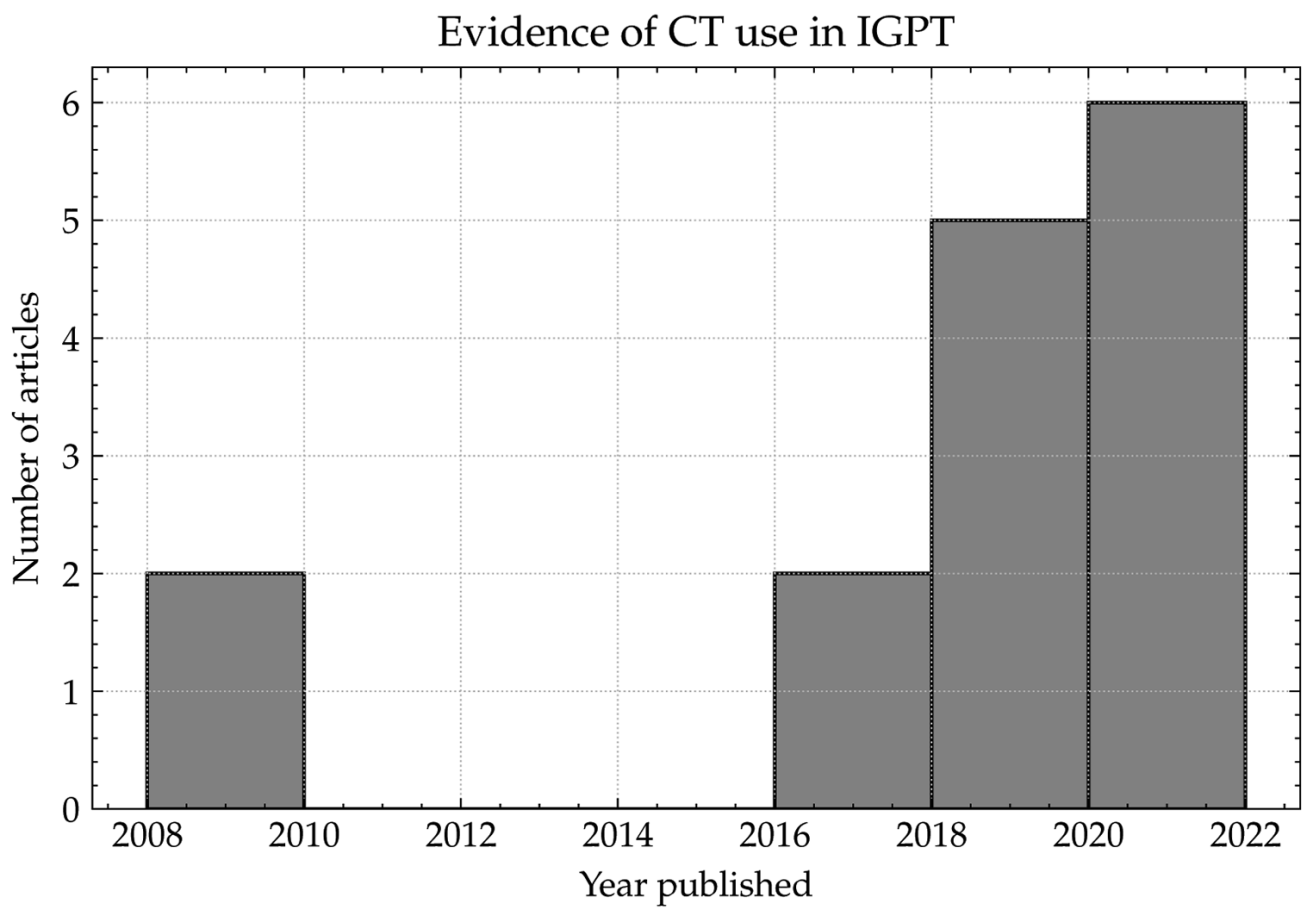
Histogram showing the number of articles over two-year periods that contained used some form of CT system used in IGPT. Note: Articles prior to 2008 were added to the 2008 bin

Table 1. summarises the publications found from the literature review in which some form of CT system was used for IGPT, while a histogram of the number of articles per two-year period (based on publication date) is shown in Fig. 2. From this group, there are three main types of CT systems in use: fixed CT, CT on rails, and mobile CT (mCT).

#### Fixed CT systems

Fixed CT systems are stationary systems that are in the treatment room or a room nearby and require the patient to be moved in and out using some form of robotic couch. In-room CT systems are typically installed close to the treatment gantry, such that the robotic treatment couch can be used to move the patient in between the treatment and imaging isocentre. These systems typically require the largest amount of floor space in the treatment room and must also be out of the way of the proton treatment system. Near-room CT systems can circumvent this issue of space by having an imaging system in a nearby separate room, however, require a specialised patient transport system for minimal patient movement between imaging and treatment isocentre. In addition, a greater degree of uncertainty must be considered due to risk of patient movement, as greater distances are covered in shifting the patient between imaging and treatment isocentres.

In 2008, Bolsi et al. [2] reported on their remote patient positioning procedure, using a near-room CT system for pre-treatment imaging for patient position verification. In their report, they used a dedicated patient transporter which was designed to be able to couple to both the CT scanner and the proton treatment itself. After remotely positioning the patient, orthogonal topograms were taken and compared to reference images from the treatment planning scan, which generated a set of translational couch corrections to minimise beam misalignment. After image analysis, the patient was transported back into the treatment room, ready for treatment after implementing the treatment couch corrections.

Other studies reported the usage of an in-room CT stationed nearby the treatment module. In 2016, Stuetzer et al. [3] presented results from a retrospective calculation of delivered proton dose to prostate cancer patient to assess their patient positioning and immobilization protocols. Retrospective dose calculations were done on daily in-room CT images that were initially acquired for assessing the stability of fiducial markers and patient anatomy after patient positioning during their treatment course. They reported slight differences with one patient receiving less dose in the iCTV (D98% = 94.6%), and two patients slightly exceeding the rectum V70Gy (< 11.3%).

In 2017 Abe [4], investigated the impact of deformable image registration (DIR) algorithm associated uncertainties on proton therapy dose accumulation using images from patients undergoing proton therapy for prostate cancer. Data from patients who had verification CT scans (either near-room or in-room) 5 min prior to their proton therapy treatment were used in their study. Dose distributions on ptCT images were warped and accumulated on the planning CT geometry using two different DIR software packages. DVH parameters for the rectum and bladder were calculated and compared between both packages. A maximum difference in V40 of 14.4% and 22.8% was reported for the rectum and bladder, respectively.

In 2019, a retrospective study from Kurz et al. [5] investigated dose-guided patient alignment using ptCT images of proton therapy patients from an in-room CT system. Initially, bony anatomy was used to align the daily ptCT images to the pCT. A series of DVH clinicals goals and their relative importance class was inputted, then using a multi-criterial optimisation method, a patient shift was generated such that it optimised dose to the patient based on the DVH clinical goals set. Kurz reported that the main impact of dose-guided patient alignment over bony-based alignment, was a reduced dose to OARs for H&N and improved coverage of target structures for prostate patients. They also found limitations in mitigating over-dosed regions in the target volume due to patient weight loss, which likely required treatment plan adaptation.

In 2021, Berthold et al. [6] aimed to validate CT-SPR methods using prompt gamma imaging (PGI) in patients. Twice weekly, patients undergoing proton therapy treatment for prostate cancer were transferred from the treatment position into an in-room DECT system and imaged immediately prior to treatment to keep track of the patient’s anatomy. Three CT-based simulations for PGI emission were generated using the DECT images with a standard HU-SPR Hounsfield look-up table (HLUT), DirectSPR which performed a voxel-wise direct SPR calculations using the DECT images, and an adapted HLUT. For each pencil-beam-scanning spot, simulated PGI emissions were then compared to experimentally measured PGI emissions with a mean range predicition accuracy of 0.0 +/- 0.5%, 0.3 +/- 0.4%, and 1.8 +/- 0.4% for DirectSPR, adapted HLUT and standard HLUT, respectively.

#### CT on-rails

CT on-rail systems are installed in the treatment room close to the treatment gantry. One primary advantage of a CT on-rails system compared to a fixed CT system, is the scanner resides in an area of the room that is out of the way until needed. When imaging is required, the treatment couch shifts the patient out of the treatment isocentre and into the imaging isocentre, then the CT system slides on the rails into the imaging location. Care is required at installation to ensure correct CT and patient couch integration, as well as ensuring no collisions during movement.

In 1999, Kamada et al. [7] reported one of the earliest usages of in-room CT on-rails for IGPT after installing an in-room vertical CT. The system was mounted to the ceiling and was used for treatment planning imaging and IGPT of patients who were treated sitting in a treatment chair. Images taken prior to treatment were used to generate treatment chair corrections for patient positioning verification. One unique feature for this system is its ability to image with the patient at treatment isocentre, which as identified by Kamada, leads to the potential for dose distribution calculations. However, its major drawback is the limitation in what treatments can be done with the patient in a seated position.

Several studies by Maeda et al. [8–10] used data from patients treated at their proton facility with IGPT. An in-room CT on-rails was used to take daily CT images of patients with prostate cancer. Treatment couch corrections were generated by first matching bony structures to the pCT, then matching the boundary between the prostate and rectum’s anterior region to the pCT. After the patient was moved back into the treatment isocentre, an additional kV x-ray is taken to confirm no change during the movement of the treatment couch from imaging isocentre to treatment isocentre.

In 2021, Nenoff et al. [11] conducted an end-to-end experimental test of a proposed daily adaptive proton therapy workflow using an anthropomorphic phantom. They used images from a dedicated in-room CT on-rails system that was used clinically for patient positioning, and rigidly registered them to a reference CT. Starting from a template plan, a new treatment plan was generated based on optimising template plan constraints, which was quickly clinically accepted based on *a priori* tolerances in DVH parameters. Nenoff reported a > 92% and > 99% gamma pass rate with a 3%/3 mm criteria when comparing measured dose to the template plan and logfile Monte Carlo dose reconstructions using the daily CT respectively, and expressed their intent to prepare for clinical commissioning of their proposed workflow.

Feng et al. [12] recently conducted a feasibility study of GPU-accelerated Monte Carlo-based online adaptive proton therapy. They report the clinical usage of CT-on-rails in their proton treatment room which is used for pre-treatment patient alignment. In their proposed workflow, ptCT and pCT images are registered using a hybrid (rigid + deformable) registration method. After approval, forward based dose calculation is performed on the new image using the initial plan. If constraints are met, continue with treatment, otherwise re-optimisation is performed using the initial constraints. They conclude the proposed workflow was efficient and effective with reoptimized plans that improve the plan quality.

#### Mobile CT systems

mCT systems have also been reported for use in IGPT. mCT systems move freely on the floor and are typically more compact than their fixed CT or CT-on-rails counterparts. This reduces the space required in the treatment room for usage of the scanner, but also for storage of the scanner as it can be stored remotely. When used for imaging, like CT-on-rails, they are typically placed near the patient allowing the use of the treatment couch to move the patient in and out of imaging isocentre. However, unlike CT-on-rails, additional care must be taken to ensure the mCT is moved into the correct position each time it is used for imaging.

In 2018, Oliver et al. [13] reported their work in commissioning the use of a mCT at their proton therapy centre. They demonstrated their methodology for generating a SPR calibration curve which enabled dosimetric equivalency between the mCT and the pCT for a variety of phantoms. They also reported usage of the mCT for ptCT imaging for patient positioning verification and alignment in six degrees-of-freedom. In addition, mCT images were used for offline dose adaptation when necessary due to clinical changes in anatomy.

Later in 2018, Sun et al. [14] investigated the feasibility of using a mCT at their proton therapy centre for IGPT. Initial work was done ensuring correct positioning of the mCT, with a reported overall localisation accuracy of < 0.5 mm. Concluding work in their report, showed use of the mCT images in pre-treatment dose calculations for adaptive planning. The mCT was used to image two patients, one H&N case the other liver, with their ptCT images rigidly registered to the pCT. DVH metrics were used to assess dose distribution to the target and OARs, with replanning done on the ptCT images where required. For the H&N case, beam range was reduced from 9.6 cm to 9.2 cm when it was identified that the maximum dose to the spinal cord increased from 9.8 to 19.6 Gy. In the liver case, V50 in the CTV was increased from 92.4 to 98.1% by adjusting the range of one beam.

In 2020, Kang et al. [15] reported their work in commissioning a compact, gantry-mounted accelerator pencil beam scanning proton system. They report the use of a mCT system, enabling 3D volumetric imaging for adaptive proton therapy for patient positioning and offline dose verification. While in 2022, Zeidan et al. [16] shared their experience of their compact proton therapy treatment system. They report that their mCT was used primarily for breast, thorax and head & neck patients for positioning verification and offline dose review. The mCT was used for other sites as requested by the physician, with an average of 3 scans per patient during their treatment. Otherwise, standard 2D kV imaging was used for daily patient setup verification.

### CBCT Systems

CBCT systems are commonly used for IGRT and naturally have been considered for use in IGPT. The primary benefit of CBCT systems is its ability to acquire images at patient isocentre with gantry mounted CBCT systems, ensuring the patient is correctly positioned immediately prior to treatment. However, due to increased scatter, the image quality of CBCT systems is not sufficient for direct dose calculation and verification [1]. In addition, the field of view (FoV) of CBCT images is typically smaller than that of the pCT, increasing the complexity of correctly registering the two images.

**Fig. 3:**
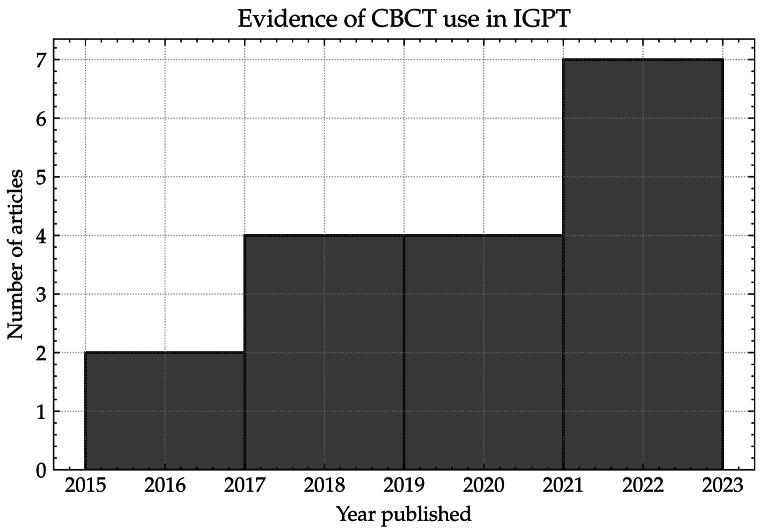
Histogram showing the number of articles over two-year periods that contained used some form of CBCT system used in IGPT

Table 2. summarises the publications found from the literature review in which some form of CBCT system was used for IGPT, and a histogram of the number of articles per two-year period (based on publication date) is shown in Fig. 3. From this group, the articles have been split into three sub-categories: Gantry installed CBCT systems, onboard CBCT systems, and other CBCT systems.

#### Gantry installed CBCT systems

Prior to 2014, gantry mounted CBCT systems were not commercially available with proton therapy units. However, due to the prevalence of CBCT imaging in conventional radiotherapy, some institutions retrospectively refitted their treatment unit’s 2D kV imaging system for 3D CBCT imaging capability.

Veiga et al. [17, 18]. used previous patient data from their proton treatment facility to assess the use of CBCT in adaptive proton therapy. A gantry mounted CBCT system was retrospectively fitted to their proton treatment unit enabling treatment isocentre imaging for patient positioning verification. However, as the original detector panel was not designed to be offset laterally, the FoV of CBCT imaging was limited. Veiga investigated the use of DIR to create virtual CT’s (vCT) by deforming the pCT to pre-treatment CBCT images and assessed their suitability for dosimetric based assessment in adaptive proton therapy. They assessed the accuracy of the vCT by comparing calculated WET values and dosimetric information to a repeat CT of the patient taken within two days of the acquired CBCT image. They report that the vCT produced similar WET and dosimetric information as the repeat CT.

In 2020, Cheon et al. [19] reported the use of an in-house developed phantom for verifying mechanical, radiation, and imaging isocentres of their proton therapy system. In their report, they described the retrospective installation of a gantry mounted CBCT which was used for patient alignment accuracy. Using their phantom, they report the largest distance difference of 0.68 mm, which was between the radiation isocentre and the imaging isocentre.

#### Onboard CBCT systems

Onboard CBCT systems are typically gantry mounted CBCT systems that were part of the design of the initial proton therapy treatment solution. Unlike retrospectively fitted systems, the CBCT system was considered in the design of the proton therapy treatment unit, allowing additional features such as a detector panel offset for larger FoV CBCT images.

In 2017, Pidikiti et al. [20] published their work in commissioning a compact pencil-beam scanning proton therapy system. They report the use of an onboard, gantry-mounted retractable x-ray system for 2D kV and CBCT imaging, which generates a correction vector from 6D image registration of acquired CBCT images to a reference image. Patient alignment was corrected through application of the computed correction vector to the robotic treatment couch.

In 2020, Chilukri et al. [21] published a report based on their experience treating younger patients (< 25 years of age) using IGPT in India. Depending on patient specific needs, 2D kV or 3D CBCT images were acquired immediately prior to treatment using the onboard, gantry mounted x-ray system. Acquired images were used to ensure treatment precision and patient positioning verification.

Work done by Thummerer et al. investigated different methods of generating synthetic CT’s (sCT) for adaptive proton therapy dose calculations using pretreatment CBCT imaging [22] and compared its suitability for head and neck patients against MR-based sCT’s [23]. In both studies, retrospective calculations were done on head and neck cancer patients who were treated at their proton therapy centre. Pretreatment CBCT images were acquired using an onboard gantry mounted CBCT system, which was used for patient positioning verification. In their study, a deep convolutional neural network (DCNN), DIR, and an analytical based correction method for generating sCT’s were compared against replanning CT’s of each patient on the same day. They report that the DCNN generated sCT had the highest image quality and resulted in accurate dose calculations. When comparing CBCT-based sCT’s to MR-based sCT’s, they report a higher average gamma pass rate (2%/2 mm criteria) for CBCT sCT’s against MR-based sCT’s (96.1% vs. 93.3%).

Recent work from Davies et al. [24] evaluated the in-room imaging workflow of their proton therapy centre. In their study, patients underwent pre-treatment CBCT imaging at a frequency dependent on their treatment site and in guidance with their institute’s guidelines. Their proton therapy unit was equipped with 2D kV imaging and an on-board nozzle mounted CBCT system. Their imaging protocol used a combination of 2D kV and CBCT imaging to ensure correct patient positioning. After initial corrections using a 2D kV system, a CBCT image was acquired to assess target volumes and OARs, using the pCT as a reference. If the setup error was within their predetermined tolerances, then no further corrections were done, and the patient was ready to treat. Otherwise, additional couch corrections are employed based on the CBCT images and a final, repeat 2D kV image was acquired to verify the movement of the couch. They found evidence suggesting a redundancy in their initial 2D kV imaging, and their imaging workflow can be streamlined by removing the initial step and rather begin at the CBCT imaging for patient positioning verification.

In 2022, a retrospective study from Stanforth et al. [25] used patient data that underwent proton therapy between December 2018 and August 2019. Daily pre-treatment CBCT images were acquired using an onboard nozzle mounted CBCT system which was used for patient positioning. The pCT was deformed to the pre-treatment CBCT images using DIR, and the initial plan was applied to the new vCT. A decision to replan was made based on anatomy volume changes and dosimetric changes. Stanforth report a replan evaluation based on the vCT has a higher specificity than that based on a replan CT.

Also in early 2022, Bondesson et al. [26]. investigated the feasibility of a 4D-vCT approach using DIR and 4D-CBCT to enable accurate dose calculation in adaptive proton therapy. CBCT images were taken of a lung phantom that executed a user-defined breathing curve using the onboard CBCT system from their proton treatment unit. As 4D-CBCT is not yet clinically available in proton therapy workflows, 3D CBCT images were used to generate a 4D-CBCT using a MA-ROOSTER workflow described in their work. The 4D-vCT was generated by registering the mid position CT to the mid position CBCT and propagating the resulting mid position vCT to a 4D-vCT using information from the 4D-CBCT. In their feasibility assessment, Bondesson conclude their results shows promise when using a 4D-CT and 4D-CBCT with the same breathing motion to generate a 4D-vCT for proton dose estimation. This was evident based on global gamma pass-rates (3%/3 mm) above 96.7% for dose thresholds of 5 Gy, 20 and 30 Gy when comparing the 4D-vCT to the initial ground truth 4D-CT.

Sheikh et al. [27] conducted a retrospective calculation of dose based on synthetic CTs (sCT) of patients treated at their proton therapy facility. sCTs were generated using deformable registration of the planning CT and CBCT images acquired from their onboard imaging system as part of their daily patient positioning verification protocol. Clinical IMPT plans were re-computed on the sCT as well as the same day quality assurance CT (qCT) and dose distributions between the two images were compared. They conclude that while sCTs were insufficient for re-planning, they are useful as they provide an approximate dosimetry of the plan without any additional imaging dose.

In 2023, Sharma et al. [28] published an article to critically appraise the current practice of IG-IMPT for craniospinal irradiation in paediatric patients at their proton therapy centre. Part of their critical appraisal covered the methodology of patient set-up error and correction strategy. The reported procedure used 2D-kV imaging for initial alignment. If setup variation was within tolerance, then onboard CBCT imaging was used to generate a 6D correction vector for patient positioning correction. If outside of tolerance, the patient’s position would be physically adjusted, and assessed with another 2D-kV image until acceptable for the CBCT imaging to proceed. This process was reduced to twice weekly after the first 5 fractions. They report that their patient positioning and verification procedure was satisfactory and robust, with all deviations falling with margin requirements.

#### Other CBCT systems

In some cases, limitations due to the design of the proton therapy unit has required that alternative methods to a gantry mounted system were required for 3D volumetric imaging. In 2015, Fattorri et al. [29] published an article describing their work in implementing and clinically validating their in-room IGPT system. A floor-installed robotic C-arm CBCT system was customised for their treatment room with out-of-treatment-isocentre imaging due to treatment room size limitations. Their clinical workflow for patient setup involved an initial setup in treatment position then, using the robotic couch, the patient was moved into imaging isocentre for 3D CBCT imaging. The acquired images were used to generate a correction vector which was implemented on the robotic couch after returning the patient to treatment isocentre.

Hua et al. [30] described the design and performance of their in-room volumetric imaging system. A ceiling mounted robotic C-arm CBCT system was installed in their proton treatment rooms for use of patient positioning verification. The ceiling mounted system had the capability of imaging both on and off treatment isocentre but required careful positioning of the treatment delivery nozzle to ensure no collisions during use. Hua reported a coincidence between the treatment and imaging isocentre of < 1 mm when imaging at treatment isocentre.

An article from Stock et al. [31] in 2018, highlighted the technological status of their ion beam therapy centre. Their reported solution for in-room 3D volumetric imaging capability was the installation of a couch mounted imaging system suitable for planar and CBCT imaging. While unable to image the patient at treatment isocentre, a calibration was generated to relate the imaging and treatment isocentre using an optical tracking camera. They report a clinical usage of CBCT imaging for patient positioning verification.

In 2021, Uh et al. [32] reported their work in training a deep neural network for CBCT based adaptive proton therapy in young adults and children. In-room CBCT images taken from a ceiling-mounted robotic C-arm CBCT system used for patient positioning verification and planning/repeat CT images of patients treated at their proton facility were used to train the model. They report a beneficial effect when combining data from adjacent anatomical sites for training the model to correct CBCT images to enable an accurate estimate of proton dose.

## Discussion

With the increased interest in proton therapy and the recent shift from treating using passively scattered protons to pencil beam scanning delivery systems, more proton therapy centres are implementing the use of in-room volumetric imaging systems for patient positioning verification. This has become much simpler now with the commercial availability of on-board CBCT imaging from proton therapy vendors, as evidenced by a large proportion of the recently published articles utilising on-board CBCT imaging. Many of the recent articles using in-room CT imaging have come from centres that had installed the system quite some time ago. For example, the mCT used in Zeidan’s [16] and Kang’s [15] work was the same mCT that Oliver [13] commissioned several years earlier. In contrast, many of the recent CBCT articles come from recently established particle centres who have simply opted to use the on-board imaging system that comes with their proton therapy solution. Since additional work and floorspace is required for CT systems, it would be unsurprising if on-board CBCT imaging begins to become the higher utilised system in the future, as it is for conventional photon therapy.

While CBCT systems may rise in popularity, their limitations for accurate dose calculations compared to CT imaging remains a problem to be solved. There has been a significant amount of research done in overcoming these issues. Early work investigated using CBCT scatter corrections and/or deformable image registration to generate a ‘virtual’ CT image for dose distribution or WET comparison with the planning CT [17, 18, 33–40]. Results indicated an accuracy of about 2% when compared to the planning CT, with some authors investigating its use as an indicator of when a replan might be necessary. More recently, work has been done investigating the use of machine learning for CBCT scatter correction and synthetic CT image creation based on CBCT images [32, 41–46], with Thummerer et al. [22] concluding that machine learning images provide proton dose calculations of similar accuracy to vCT’s based on DIR, while having a higher image quality. The ability to perform dose calculations based on CBCT imaging will be a large step towards toward the goal for online adaptive proton therapy.

As previously mentioned, one of the drawbacks of using an in-room CT system for IGPT is the additional uncertainty associated with moving the patient between treatment and imaging isocentre. Recent work from Nestueruk et al. [47] evaluated the efficacy of CT-on-rails against in-room CBCT imaging for daily adaptive proton therapy of head and neck (H&N) cancers. Dose was calculated on vCT’s which were generated using the pCT and daily CBCT images available from previous H&N patients. CT-on-rail associated uncertainties were simulated by applying a randomly sampled gaussian offset in the posterior-anterior, left-right, and inferior-superior axes for three different uncertainty values. DVH metrics were evaluated for each adaptive treatment scenario. The authors found that the uncertainty associated with couch-motion-induced patient movement had no effect on dosimetric adaptation efficacy if it was below a conservative value of 2 mm. The authors also reported that the available data on patient position uncertainty when using a CT-on-rails has an accuracy and precision of 0.3 and 1.6 mm respectively, well below the 2 mm limit. Based on their results, CT-on-rails may be an excellent alternative to CBCT imaging for dosimetry based IGPT due to the additional advantages of fan-beam CT over CBCT imaging.

Other novel imaging modalities have also been suggested in literature as a potential solution for enabling in treatment isocentre volumetric imaging. Several studies have suggested an improvement in dose calculations using dual-energy CBCT and CT images [48–52]. This improvement primarily comes from the reduced uncertainty is SPR estimation from DE imaging, but DE images also offer an improvement in contrast and metal artifact reduction. Future systems are looking into 4D imaging capabilities for addressing intra-fractional shifts.

With the recent development of an MR-linac, MR-guided proton therapy has also been actively investigated for in-room volumetric imaging. In addition to providing volumetric images at no additional dose cost, MR images also provide superior soft tissue contrast compared to photon imaging. However, unlike photons, protons are charged particles and in the presence of a magnetic field can be deflected. This interplay is one of the challenges in integrating an MR-imaging system with a proton therapy unit. Recent review articles [53, 54] have highlighted some promising proof of concept studies, but further work is required before it can be introduced clinically.

Proton CT imaging is another system that has been investigated for treatment planning and patient alignment. It offers several advantages over x-ray CT imaging, including a lower dose associated with the scan, absence of x-ray imaging artefacts and improved SPR measurements for dose calculations. There have been several prototype proton CT systems reported in literature [55–58], each reporting some technical challenges that need to be addressed. However, one of the biggest challenges for proton CT imaging is the decrease in spatial resolution due to multiple Coulomb scattering. Methods such as the most likely path formulation [59] have attempted to account for this effect, but further work is required if proton CT images are to achieve the same spatial resolution as its x-ray counterpart.

Finally in 2017, Zhu et al. [60] reported simulation results using an Eu-155 based DECBCT system for SPR measurements. In addition to the previous benefits mentioned from a DE-CBCT image, it was reported that using Eu-155 as the imaging source offered the capability of scatter removal and temporally and spatially coincident imaging due to the two primary gamma emissions at 86.5 and 105.3 keV. However, based on their calculations a 6 TBq Eu-155 source is required to obtain an image with a 1 revolution/min rotation speed. Obtaining an Eu-155 source with this activity will likely be a challenge that will need to be addressed.

As in-room volumetric imaging is still relatively new in proton therapy, it seems the frequency of the use of this tool can vary between centres. As a recent example, Sheikh et al. [27] report the use of daily CBCT imaging for paediatric patient positioning verification, while Sharma et al. [28] report a reduced amount of CBCT imaging, with daily imaging in the first 5 fractions and then only twice weekly after that for their paediatric patients. Part of this reason could be due the concern of additional imaging dose to the patient when moving from conventional 2D kV orthogonal imaging to volumetric imaging. It is important that this additional cost should be weighed against the potential benefit when deciding on the frequency of volumetric imaging.

## Conclusion

With the increased commercial availability of on-board volumetric imaging systems, there is a notable shift in proton therapy centres towards 3D volumetric imaging for patient alignment verification. Many systems are being actively researched, but at this moment it looks like CBCT imaging will become a commonly available tool in proton therapy centres due to the commercial availability of on-board CBCT imaging with proton therapy solutions.
