# Correction to: A systematic review of volumetric image guidance in proton therapy

**DOI:** 10.1007/s13246-023-01301-z

**Published:** 2023-07-20

**Authors:** Mitchell Herrick, Scott Penfold, Alexandre Santos, Kevin Hickson

**Affiliations:** 1 Department of Radiation Oncology, Royal Adelaide Hospital, Adelaide, Australia; 2 Department of Physics, University of Adelaide, Adelaide, Australia; 3 Australian Bragg Centre for Proton Therapy and Research, University of Adelaide, Adelaide, Australia; 4 SA Medical Imaging, Adelaide, Australia; 5 University of South Australia, Allied Health & Human Performance, Adelaide, Australia

**Correction to: Physical and Engineering Sciences in Medicine**

10.1007/s13246-023-01294-9

In this article the graphics relating to the captions of Figures 2 and 3 had been interchanged and six references were not included in the reference list, leading to incorrect reference numbers in the text. The correct figures and missing references are shown below.

The original article has been corrected.

**Fig. 2:**
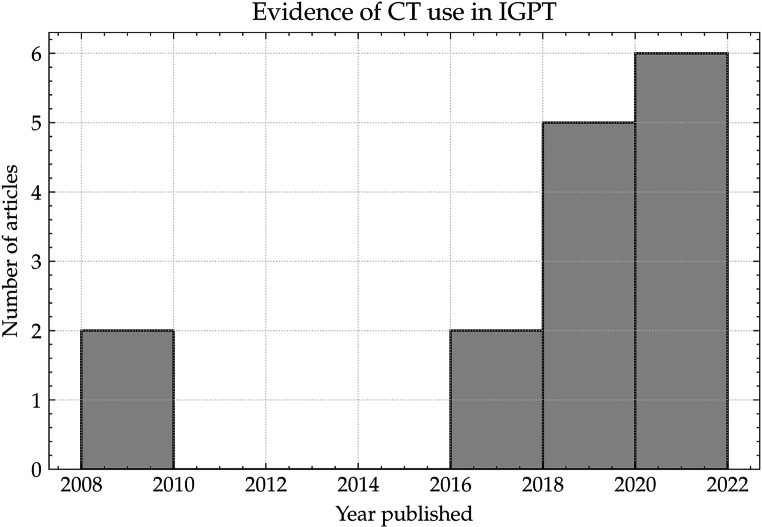
Histogram showing the number of articles over two-year periods that contained used some form of CT system used in IGPT. Note: Articles prior to 2008 were added to the 2008 bin

**Fig. 3:**
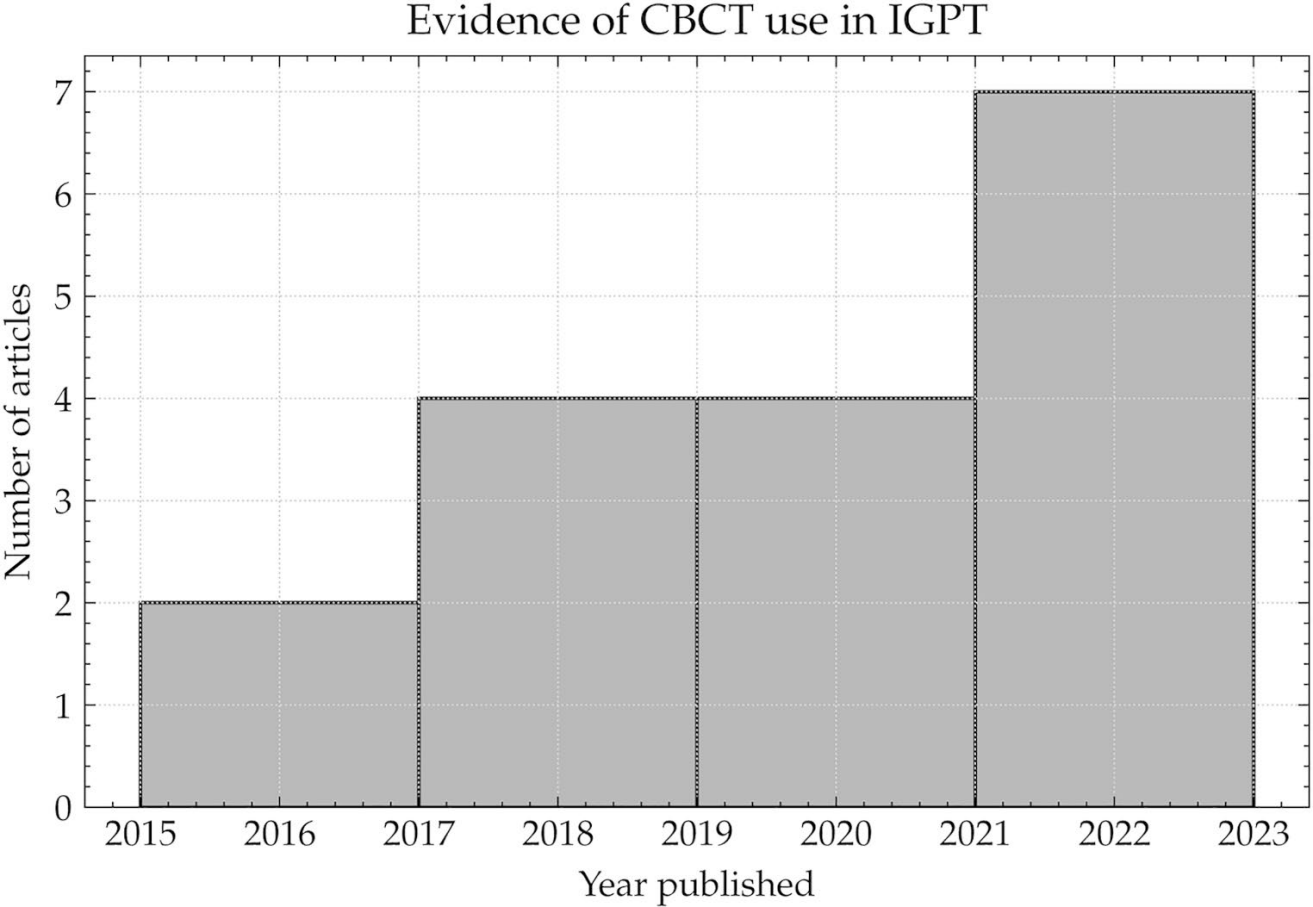
Histogram showing the number of articles over two-year periods that contained used some form of CBCT system used in IGPT
